# Cytochrome P450 Omega-Hydroxylase 4a14 Attenuates Cholestatic Liver Fibrosis

**DOI:** 10.3389/fphys.2021.688259

**Published:** 2021-05-31

**Authors:** Sha Li, Chenghai Wang, Xiaxia Zhang, Wen Su

**Affiliations:** ^1^Medical College, Hebei University of Engineering, Handan, China; ^2^Hebei Key Laboratory of Applied Basic Research of Blood Purification, Affiliated Hospital of Hebei Engineering University, Handan, China; ^3^Health Science Center, Shenzhen University, Shenzhen, China; ^4^Department of Gastroenterology and Hepatology, Handan Central Hospital, Handan, China; ^5^Shenzhen Key Laboratory of Metabolism and Cardiovascular Homeostasis, Shenzhen, China

**Keywords:** Cyp4a14, bile duct ligation, liver injury, liver fibrosis, cholestasis

## Abstract

**Background:**

Cholestasis is a pathological condition involving obstruction of bile secretion and excretion that results in hepatotoxicity, inflammation, fibrosis, cirrhosis, and eventually liver failure. Common bile duct ligation (BDL) model is a well-established murine model to mimic cholestatic liver fibrosis. We previously reported that cytochrome P450 omega-hydroxylase 4a14 (Cyp4a14) plays an important role in the pathogenesis of non-alcoholic fatty liver disease (NAFLD)-related fibrosis. The goal of this study was to determine the role of Cyp4a14 in cholestatic-induced liver fibrosis.

**Methods:**

C57BL/6 mice were subjected to BDL for 14 days, and Cyp4a14 mRNA and protein levels were examined and compared with those of the sham group. Cyp4a14 knockout mice and adeno-associated virus (AAV)-mediated overexpression of Cyp4a14 in C57BL/6 mice underwent BDL and liver histology, and key fibrosis markers were examined.

**Results:**

Both hepatic Cyp4a14 mRNA and protein levels were markedly reduced in BDL liver compared with the time-matched sham group. Cyp4a14 gene-deficient mice aggravates whereas its overexpression alleviates BDL-induced hepatic fibrosis, which were determined by liver function, liver histology, and levels of key fibrotic markers including α-smooth muscle actin (α-SMA), transforming growth factor-β1 (TGF-β1), and collagen 1a2 (Col1a2).

**Conclusion:**

Cyp4a14 exerts a contrasting role in different hepatic fibrosis models. Strategies that enhance Cyp4a14 activity may be potential strategies to cholestatic related liver fibrosis.

## Introduction

Liver fibrosis is a progressive pathological process of most chronic liver diseases with a global prevalence of more than 2%. It is characterized by excess accumulation of extracellular matrix (ECM; Aydin and Akcali, 2018). If left untreated, liver fibrosis will lead to cirrhosis and ultimately end-stage liver failure and death. Almost all the chronic liver diseases can cause liver fibrosis, such as alcoholism, chronic viral hepatitis [hepatitis B (HBV) and hepatitis C (HCV)], non-alcoholic steatohepatitis, obesity, autoimmunity hepatitis, parasitic diseases (e.g., schistosomiasis), metabolic disorders, biliary tract diseases, long-term exposure to toxicity and chemical substances, and drug-induced chronic liver disease (Toosi, 2015). Because of the complicated mechanisms in the progression of liver fibrosis (Parola and Pinzani, 2019; Seki and Brenner, 2015), even though we have made considerable progress in the prevention and treatment of liver fibrosis, treatment of liver fibrosis remains limited (Altamirano-Barrera et al., 2017; Roehlen et al., 2020).

Cholestatic liver fibrosis is caused by the gradual destruction of the bile duct, the blockade of bile acid outflow, and the activation of the pro-inflammatory process, leading to the damage of bile duct cells and liver cells (Santiago et al., 2018). Primary biliary cholangitis (PBC) and primary sclerosing cholangitis (PSC) are the two most common chronic cholestatic liver fibrosis (Gulamhusein and Hirschfield, 2020; Vesterhus and Karlsen, 2020). However, the underlying mechanism remains obscure, the mechanistic basis of which the fibrotic response is an area of current investigation.

Cytochrome P450 omega-hydroxylase 4a14 (Cyp4a14) belongs to the enzyme family of Cyp450, which are NADPH monooxygenases responsible for catalyzing a variety of substrates. Cyp4a14 was firstly cloned from mice in 1997 and mainly exists in the liver, kidney, and blood vessel (Heng et al., 1997). The main function of Cyp4a14 is to catalyze ω-hydroxylation of medium-chain fatty acids and arachidonic acid (AA; Xu et al., 2011). Peroxisome proliferator-activated receptor (PPAR) α serves as a biological lipid sensor, and Cyp4a14 is an established target gene of PPARα, always thought to be involved in lipid metabolism (Hsu et al., 2007; Kroetz et al., 1998). Our previous data and others revealed a critical role of Cyp4a14 in the pathogenesis of non-alcoholic fatty liver disease (NAFLD) and related fibrosis (Gilani et al., 2018; Zhang et al., 2017).

In the current study, we checked the expression level of Cyp4a14 in cholestatic related liver fibrosis and systematically studied the role of Cyp4a14 in bile duct ligation (BDL) model by using knockout and adeno-associated virus (AAV)-mediated overexpression technology. Notably, we observed an opposite effect of Cyp4a14 in BDL model. Our results indicate a different but also important role of Cyp4a14 in the pathogenesis of cholestatic liver fibrosis.

## Materials and Methods

### Animals and Treatments

Wild-type (WT) and Cyp4a14 gene knockout (Cyp4a14^–/–^) mice on a 129/SvJ background originally generated by Dr. J. Capdevila, Vanderbilt University, were kindly provided by Pro. YF Guan (Dalian Medical University, China). Male C57Bl/6 mice (6 weeks old) were purchased from the Institute of Medical Laboratory Animal Center, Guangdong, China. Animals were housed under a 12:12-h light/dark cycle and permitted *ad libitum* consumption of water and diet. All experimental procedures were approved by the Animal Experimentation Committee of Shenzhen University Health Science Center. For BDL model, C57BL/6 mice were randomly divided into two groups (sham operation and surgery group), and all surgeries were performed between 9:00 AM and 11:00 AM to minimize the variation. Then we performed BDL in Cyp4a14^–/–^ mice (Cyp4a14^–/–^ + BDL, *n* = 8) and its WT (WT + Sham, *n* = 7; WT + BDL, *n* = 8). For overexpression experiments, C57BL/6J mice were randomly divided into three groups: sham operation (Sham, *n* = 7), BDL operation in combination with injection of AAV-control (AAV-control + BDL, n = 8), and BDL operation in combination with injection of AAV-pGFAP-Cyp4a14 (AAV-Cyp4a14 + BDL, *n* = 8). The AAV was injected via the tail vein 4 weeks before the surgery (1 × 10^11^ pfu per mouse). Two weeks after BDL, all mice were sacrificed under isoflurane anesthesia. Blood were collected from the vena cava to isolate serum. The left lateral liver lobe was fixed in 4% paraformaldehyde, then changed in 20% sucrose solution, and embedded in paraffin. The remaining livers were snap frozen in liquid nitrogen.

### Immunohistochemistry

Mouse livers were fixed, dehydrated, and embedded in paraffin wax. Fixed liver sections (5 μm) were incubated with an anti-α-smooth muscle actin (anti-α-SMA) antibody (1:200), an anti-Col1a2 antibody (1:500), or an anti-smad3 antibody (1:100) overnight at 4°C and then with a polyperoxidase-conjugated goat anti-rabbit IgG (Zhongshan Golden Bridge, Beijing, China) for 30 min at 37°C. The slides were counterstained with hematoxylin. ImageJ was used to semi-quantify the H-score of immunohistochemistry (IHC) staining.

### Reagents and Chemicals

Kits for determining serum alanine aminotransferase (ALT) and aspartate aminotransferase (AST) were obtained from Nanjing Jiancheng Bioengineering Institute (Nanjing, China). Total collagen content was tested by measuring the amount of hydroxyproline (Hyp) in liver tissue using Hyp assay kit obtained from Nanjing Jiancheng Bioengineering Institute (Nanjing, China). The liver sections imbedded in paraffin were cut (5 μm) and stained with hematoxylin–eosin (H&E), Sirius Red, and Masson’s trichrome. Image J was used to determine the Sirius Red-positive area. The macroscopic examination was blindly carried out by two independent observers. AAV (type9)-control and AAV-promoter of glial fibrillary acidic protein (pGFAP)-Cyp4a14 (type9, contract number HH20181228LQJ-AAV01) were purchased from Hanbio, Shanghai, China.

### Quantitative RT-PCR Analysis

The analysis method was performed as described previously (Zhang et al., 2017). Total RNA was extracted from the frozen liver tissues by using a High Purity Total RNA Extraction Kit (BioTeke, Beijing, China). Five micrograms of total RNA from each sample was reverse-transcribed into complementary DNAs (cDNAs). Each cDNA sample was diluted 1:100, and 4 μl was used as a template per PCR (Thermo Fisher Scientific, Massachusetts, CA, United States). The quantitative PCR was performed on an Agilent Mx3000P PCR System (Agilent Technologies, Santa Clara, CA) using the TransStart Top Green qPCR SuperMix (TransGen, Beijing, China). Expression levels of the target genes were normalized against an endogenous reference gene, glyceraldehyde 3-phosphate dehydrogenase (GAPDH). For each sample and each gene, PCR was carried out in duplicate and repeated for a few times. The specific primer sequences are listed in Table 1.

**TABLE 1:** Sequences of primers used for real-time quantitative PCR.

**Gene**	**Forward primer sequence (5′–3′)**	**Reverse primer sequence (5′–3′)**	**Reaction temperature (°C)**
Cyp4a14	TGAATTGCTGCCAGATCCCAC	GTTCAGTGGCTGGTCAGAGTT	59
Atca2	CTGACAGAGGCACCACTGAA	CATCTCCAGAGTCCAGCACA	59
Tgfb1	TGACGTCACTGGAGTTGTACGG	GGTTCATGTCATGGATGGTGC	59
Col1a1	CAATGCAATGAAGAACTGGACTGT	TCCTACATCTTCTGAGTTTGGTGA	59
Col1a2	GCAGGGTTCCAACGATGTTG	GCAGCCATCGACTAGGACAGA	59
Mmp2	CAAGTTCCCCGGCGATGTC	TTCTGGTCAAGGTCACCTGTC	59
Mmp9	CTGGACAGCCAGACACTAAAG	CTCGCGGCAAGTCTTCAGAG	59
Timp1	GCAACTCGGACCTGGTCATAA	CGGCCCGTGATGAGAAACT	59
Tgfbr1	GGCGAAGGCATTACAGTGTT	TGCACATACAAATGGCCTGT	59
Smad3	CTGGGCCTACTGTCCAATGT	GCAGCAAATTCCTGGTTGTT	59
Pparα	ATGCCAGTACTGCCGTTTTC	GGCCTTGACCTTGTTCATGT	59
GAPDH	AGAACATCATCCCTGCATCC	TTGTCATTGAGAGCAATGCC	59

### Western Blot Analysis of Hepatic Proteins

The analysis method was performed as described previously (Zhang et al., 2017). To determine the expression levels of selected proteins, 80 μg of liver protein was separated by 10% sodium dodecyl sulfate (SDS) gel. Western blot analysis was performed as described using the antibodies including anti-GAPDH (1:3,000, abs830030, Absin), anti-Cyp4a14 (1:3,000, E10821, ABclonal), anti-Col1a2 (1:1,000, BS-1530), and anti-α-SMA (1:1,000, SIGMA-A2547, Sigma). Immunoblot was performed, and the membrane was developed with enhanced chemiluminescence.

### Statistical Analysis

Statistical evaluation was performed by Student’s *t* test when only two value sets were compared and one-way ANOVA followed by Bonferroni’s test when the data involved multiple groups. *p* < 0.05 was considered statistically significant.

## Results

### Hepatic Cyp4a14 Expression Is Reduced in Cholestatic Liver

**FIGURE 1:**
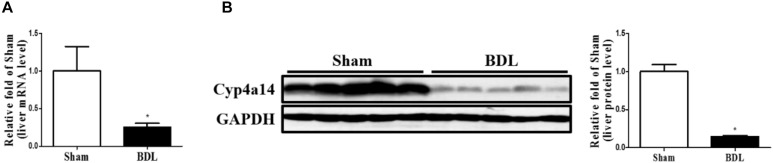
The expression of Cyp4a14 was decreased in the bile duct ligation (BDL)-induced fibrotic liver. **(A)** Real-time PCR assay demonstrating that Cyp4a14 mRNA expression was significantly decreased in BDL mice. **(B)** Western blot analysis showing a significant decrease in hepatic Cyp4a14 protein expression in BDL mice. *n* = 5, *p < 0.05, Sham and BDL.

To explore the biological relevance between Cyp4a14 and cholestatic liver fibrosis, we first examined Cyp4a14 expression levels in both sham and BDL mice liver. As shown in Figure 1, 14 days after BDL, hepatic Cyp4a14 mRNA levels were downregulated markedly by 75% compared with the sham-operated mice (Figure 1A). In accordance with that, Cyp4a14 protein expression was reduced to 15% in the sham-operated mice (Figure 1B). The decrease of Cyp4a14 in BDL model is opposite with NAFLD murine model such as high fat diet (HFD)-treated mice, db/db mice, and methionine and choline-deficient (MCD) diet-treated mice (Zhang et al., 2017). The above findings suggest that Cyp4a14 may play a different role in the pathogenesis of cholestatic liver fibrosis.

### Ablation of Cyp4a14 Gene Aggravates Bile Duct Ligation-Induced Liver Fibrosis

**FIGURE 2:**
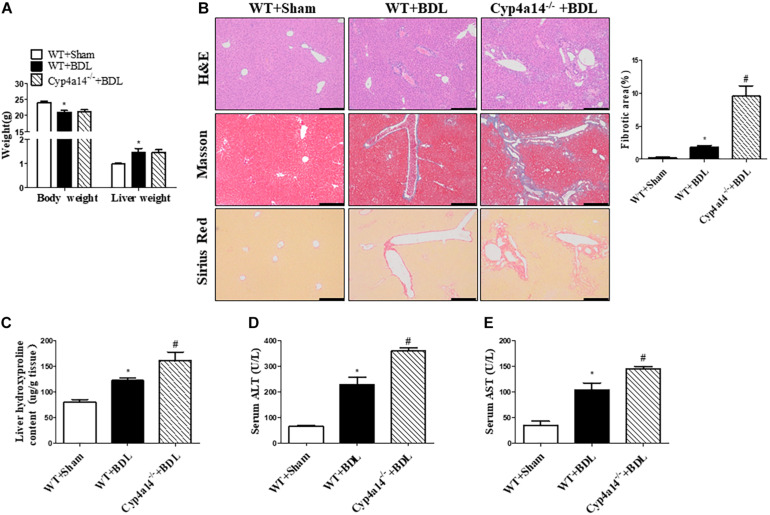
Cyp4a14 deficiency aggravated bile duct ligation (BDL)-induced hepatic fibrosis. Wild-type (WT) and Cyp4a14^–/–^ mice were subjected to BDL for 14 days. **(A)** Body weight and liver weight. **(B)** H&E, Masson, and Sirius Red staining of the mouse liver tissues. **(C)** Hepatic collagen content was measured by biochemical determination of hydroxyproline (Hyp). Serum alanine aminotransferase (ALT) **(D)** and aspartate aminotransferase (AST) **(E)** levels of WT and Cyp4a14^–/–^ mice. *n* = 6–7. *p < 0.05, WT + Sham and WT + BDL. ^#^*p* < 0.05, WT + BDL and Cyp4a14^–/–^ + BDL. Scale bar = 100 μm.

On the basis of the decrease of Cyp4a14 expression in fibrotic liver, we next examined the effects of Cyp4a14 ablation on BDL-induced liver fibrosis. BDL was performed in Cyp4a14^–/–^ mice and respective WT controls. At day 14 after surgery, the body weight was significantly decreased, and liver weight was significantly increased in the WT + BDL group compared with the WT + Sham group (Figure 2A), which was consistent with previous research (Lee et al., 2021). There was no difference between the WT + BDL group and the Cyp4a14^–/–^ + BDL group (Figure 2A). We used Masson and Sirius Red staining to determine the formation of fibrillar collagen as a measure of liver fibrosis. As Figure 2B shows, BDL leads to the proliferation of the biliary duct with matrix deposition in the periductal and lobular areas. Next, the effects of Cyp4a14 in BDL-induced fibrosis were compared in liver fibrosis between WT and Cyp4a14^–/–^ mice. Compared with the WT + BDL group, the Cyp4a14^–/–^ + BDL group showed a marked increase in fibrotic area as evidenced by the Masson and Sirius Red staining (Figure 2B). We also tested the liver total collagen content by measuring the amount of Hyp in liver tissue. In line with the histological analysis, Hyp level is also increased in the Cyp4a14^–/–^ + BDL group compared with WT + BDL group (Figure 2C). Moreover, serum ALT and AST were significantly higher in the Cyp4a14^–/–^ + BDL group compared with the WT + BDL group, which revealed an increased liver injury (Figures 2D,E). These data indicate increased liver damage after BDL in the Cyp4a14^–/–^ + BDL group.

### Cyp4a14 Ablation Enhanced α-Smooth Muscle Actin, Col1a2, and smad3 Expression in Bile Duct Ligation Mice

**FIGURE 3:**
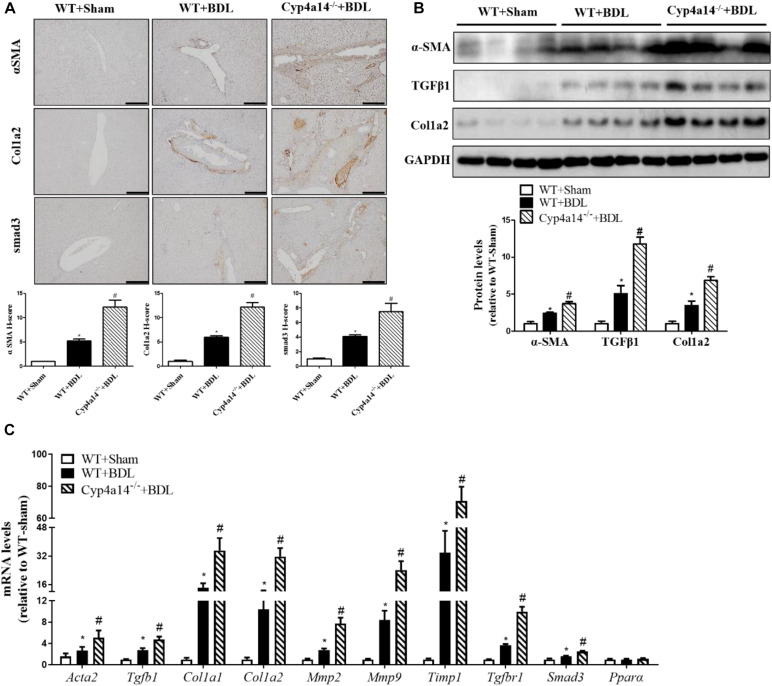
Cyp4a14 ablation enhanced α-smooth muscle actin (α-SMA), Col1a2, and smad3 expression in bile duct ligation (BDL) mice. **(A)** Cyp4a14 gene ablation markedly exacerbated BDL-induced α-SMA, Col1a2, and smad3 protein expression in the livers as assessed by an immunostaining analysis. **(B)** Western blot assay showing the protein levels of α-SMA, TGF-β1, and Col1a2. **(C)** Quantitative RT-PCR analysis showing the mRNA levels of *Acta2*, *Tgfb1*, *Col1a1*, *Col1a2*, *Mmp2*, *Mmp9*, *Timp1*, *TgfbR1*, *Smad3*, and *Ppar*α. *n* = 6–7. *p < 0.05, WT + Sham and WT + BDL. ^#^*p* < 0.05, WT + BDL and Cyp4a14^–/–^ + BDL. Scale bar = 100 μm.

We next examined a series of pro-fibrotic factors. Hepatic stellate cells (HSCs) play pivotal roles in the pathological development of liver fibrosis. α-SMA acts as a marker of activated HSCs. To evaluate the involvement of Cyp4a14 in HSC activation in cholestatic liver fibrosis, expression of α-SMA was examined via both IHC assay and western blot analysis. As expected, α-SMA staining and protein level were markedly increased in the WT-BDL group mice compared with the sham group, and about two-fold higher expression was observed in the Cyp4a14^–/–^ + BDL group mice compared with the WT-BDL group (Figures 3A,B). In accordance with that, other key pro-fibrotic factors including TGF-β1, Col1a2, and smad3 were also increased in the Cyp4a14^–/–^ + BDL group compared with the WT + BDL group (Figures 3A,B). In addition, BDL-induced gene expression of pro-fibrotic and fibrosis-related genes (*Acta2*, *Tgfb1*, *Col1a1*, *Col1a2*, *Mmp2*, *Mmp9*, *Timp1*, *TgfbR1*, *Smad3*, and *Ppar*α) was markedly worsened in the Cyp4a14^–/–^ + BDL group compared with the WT + BDL group (Figure 3C). Together, the above data showed that liver injury and fibrosis were more prominent in Cyp4a14^–/–^ livers after BDL.

### Hepatic Cyp4a14 Overexpression Attenuates Bile Duct Ligation-Induced Liver Fibrosis

**FIGURE 4:**
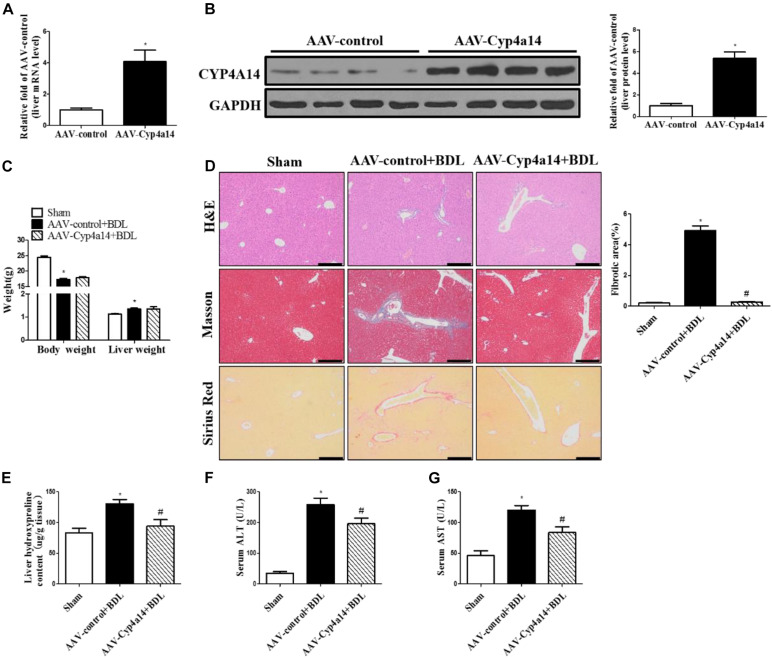
Hepatic Cyp4a14 overexpression ameliorated bile duct ligation (BDL)-induced hepatic fibrosis. Adeno-associated virus (AAV)-control and AAV-Cyp4a14 mice were subjected to BDL for 14 days. **(A)** Body weight and liver weight. **(B)** H&E, Masson, and Sirius Red staining of the mouse liver tissues. **(C)** Hepatic collagen content was measured by biochemical determination of hydroxyproline (Hyp). Serum alanine aminotransferase (ALT) **(D)** and aspartate aminotransferase (AST) **(E)** levels of AAV-control and AAV-Cyp4a14 mice. *n* = 6–7. *p < 0.05, Sham and AAV-control + BDL. ^#^*p* < 0.05, AAV-control + BDL and AAV-Cyp4a14 + BDL. Scale bar = 100 μm.

After showing that Cyp4a14 was an important player in liver fibrosis after BDL, we then addressed the question whether overexpression could rescue this effect. HSC activation is the center process in BDL-induced fibrosis. We hypothesized that Cyp4a14 may play an important role in the HSC activation. To explore the exact role of Cyp4a14 in HSC activation, we constructed a GFAP-promoter driven AAV9-Cyp4a14. We overexpressed Cyp4a14 in the HSCs of WT mice via tail vein injection 2 weeks before BDL surgery. Cyp4a14 overexpression was succeeded and validated by mRNA and protein levels by four- to seven-fold (Figures 4A,B). The body weight was also obviously decreased while liver weight was increased in the AAV-control + BDL group compared with the sham group (Figure 4C). There was no difference between the AAV-control + BDL group and the AAV-Cyp4a14 group (Figure 4C). Masson and Sirius Red staining revealed a significant decrease in fibrotic area in the AAV-Cyp4a14 + BDL group compared with the AAV-control + BDL group (Figure 4D). In line with the histological analysis, liver Hyp content is also improved in the AAV-Cyp4a14 + BDL group compared with the AAV-control + BDL group (Figure 4E). In addition, serum ALT and AST were significantly lower in the AAV-Cyp4a14 + BDL group compared with the AAV-control + BDL group (Figures 4F,G). These together confirmed that BDL-induced liver damage was alleviated after HSC Cypa14 overexpression.

### Hepatic Cyp4a14 Overexpression Inhibited α-Smooth Muscle Actin, Col1a2, and smad3 Expression in Bile Duct Ligation Mice

**FIGURE 5:**
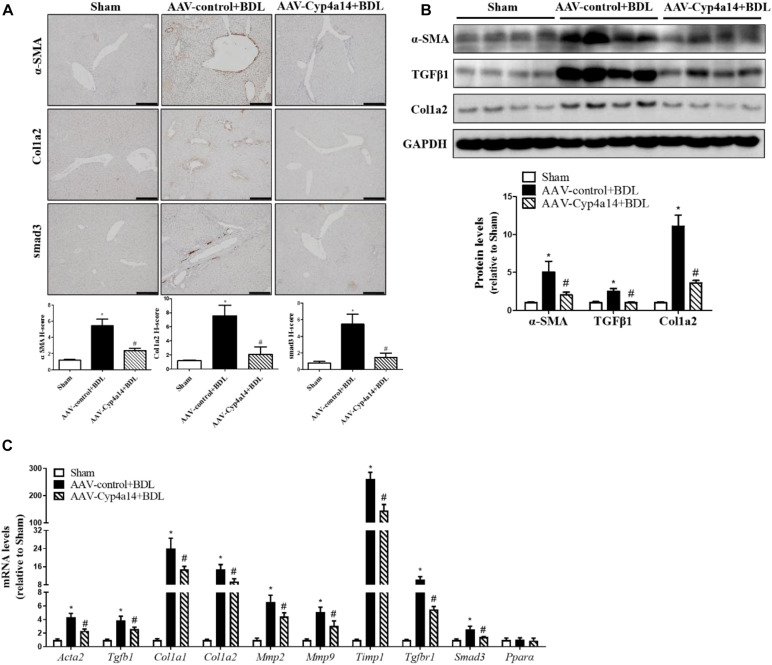
Hepatic Cyp4a14 overexpression inhibited α-smooth muscle actin (α-SMA), Col1a2, and smad3 expression in bile duct ligation (BDL) mice. **(A)** Cyp4a14 gene overexpression obviously attenuated BDL-induced α-SMA, Col1a2, and smad3 protein expression in the livers as assessed by an immunostaining analysis. **(B)** Western blot assay showing the protein levels of α-SMA, TGF-β1, and Col1a2. **(C)** Quantitative RT-PCR analysis showing the mRNA levels of *Acta2*, *Tgfb1*, *Col1a1*, *Col1a2*, *Mmp2*, *Mmp9*, *Timp1*, *TgfbR1*, *Smad3*, and *Ppar*α. *n* = 6–7. *p < 0.05, Sham and AAV-control + BDL. ^#^*p* < 0.05, AAV-control + BDL and AAV-Cyp4a14 + BDL. Scale bar = 100 μm.

We also examined the protein expression of α-SMA, TGF-β1, Col1a2, and smad3. In accordance with the reduction of collagen deposition seen by the morphological changes, all the pro-fibrotic factors were significantly decreased in the AAV-Cyp4a14 + BDL group compared with the AAV-control + BDL group (Figures 5A,B). And the hepatic expression of pro-fibrotic and fibrosis-related gene (*Acta2*, *Tgfb1*, *Col1a1*, *Col1a2*, *Mmp2*, *Mmp9*, *Timp1*, *TgfbR1*, *Smad3*, and *Ppar*α) mRNAs were also decreased in the AAV-Cyp4a14 + BDL group compared with the AAV-control + BDL group (Figure 5C). These results suggest that the increase of Cyp4a14 in HSCs is critical for protection of BDL-induced liver fibrosis.

## Discussion

Hepatic fibrosis is a dynamic process in which the production and dissolution of ECM are unbalanced (Aydin and Akcali, 2018). The development of liver fibrosis is coordinated by a variety of cell types, including parenchymal and non-parenchymal cells.

We had previously demonstrated that Cyp4a14 is involved in the pathogenesis of liver fibrosis and kidney fibrosis (Zhang et al., 2017; Zhou et al., 2018). Our previous study showed that Cyp4a14 is activated in mice suffering from MCD-induced liver fibrosis, and Cyp4a14^–/–^ mice could attenuate the MCD-induced liver fibrosis, accompanied by reduced hepatic FAT/CD36 expression (Zhang et al., 2017). In the present study, we aim to explore the role of Cyp4a14 in BDL-induced cholestatic liver fibrosis. To our surprise, hepatic Cyp4a14 expression was significantly decreased in BDL liver, which is opposite with NAFLD-related fibrosis. Cyp4a14 ablation mice suffering from BDL exhibited a markedly aggravated HSC activation (shown by α-SMA mRNA and protein level) and collagen deposition (shown by Masson and Sirius Red staining). The evidence revealed that Cyp4a14 might regulate activation of HSCs to play a role in cholestatic liver fibrosis. Thus, we propose a critical role of Cyp4a14 in HSCs. We overexpressed AAV9-Cyp4a14 driven by an HSC-specific promoter GFAP to overexpress Cyp4a14 in HSCs. We found that overexpression of Cyp4a14 in the HSCs markedly rescued BDL-induced liver fibrosis. HSC activation was significantly inhibited (shown by α-SMA mRNA and protein level) and collagen deposition ameliorated (shown by Masson and Sirius Red staining). TGF-β1 plays an important role in HSC activation (Dewidar et al., 2019). Our study shows an obviously reduction of liver TGF-β1 upon Cyp4a14 overexpression mice, which is increased in Cyp4a14^–/–^ mouse livers. Additionally, the expression levels of *Acta2*, *Tgfb1*, *Col1a1*, *Col1a2*, *Mmp2*, *Mmp9*, *Timp1*, *TgfbR1*, *Smad3*, and *Ppar*α in BDL-mouse livers were downregulated by Cyp4a14 overexpression and upregulated by Cyp4a14 ablation. These results together indicate that Cyp4a14 might have a different role in hepatocyte and stellate cell during fibrosis.

Bile duct ligation-induced cholestatic liver fibrosis model is commonly used to mimic human hepatic fibrosis, and many researchers are seeking anti-fibrotic therapeutics by studying the etiology and pathology (Liedtke et al., 2013; Tag et al., 2015). BDL leads to the stasis of bile acids in the liver. Excess accumulation of toxic bile salts in hepatocytes results in inflammatory reactions, hepatocyte necrosis, and peri-ductular fibrosis (Frissen et al., 2020). HSC activation plays a pivotal role in the development of liver fibrosis. Farnesoid X Receptor (FXR) is a promising target in clinical trials for the treatment of liver fibrosis. FXR agonists such as obeticholic acid (OCA) have been approved by the Food and Drug Administration (FDA) and are currently widely used for treatment of PBC (Chapman and Lynch, 2020). However, the response rate of OCA treatment to NASH-induced liver fibrosis is only 23%, and common adverse effects such as pruritus and unfavorable changes in lipid profile led OCA to be declined by the FDA for treatment of NASH (Younossi et al., 2019). The discrepancy of FXR in PBC and NASH might reflect the complexity of the HSC activation process (Dewidar et al., 2019; Khomich et al., 2019). The discrepancy of Cyp4a14 in NASH and PBC animal model is very similar to FXR. FXR activation by OCA or GW4064 increased expression of Pparα, Cpt1α, and Cyp4a14 in HFD mice (Gai et al., 2016). The nuclear receptor SHP mediates inhibition of HSCs by FXR and protects against liver fibrosis (Fiorucci et al., 2004). It is reported that SHP upregulates Cyp4a14 through inhibition of Rev-erbα, a transcriptional repressor the Cyp4a14 (Zhang et al., 2018). Thus, it is possible that Cyp4a14 might mediate FXR activation benefits indirectly in OCA-treated PBC. In addition, different causes lead to the different pathogenesis of liver fibrosis and may provide a basis for the new treatment methods (Gressner et al., 2007; Roehlen et al., 2020).

There are some limitations to our study. First, primary hepatocytes and stellate cell experiment with silencing or overexpressing Cyp4a14 would be helpful to elucidate how exactly Cyp4a14 affect BDL-induced fibrosis. Second, animal experiments including hepatocyte specific Cyp4a14 overexpression and HSC specific Cyp4a14 deletion in BDL model should be checked to rule out the contribution and role of Cyp4a14. Furthermore, whether Cyp4a14 affects bile acid pool or FXR signaling might give some clues to explain our current data. More work needs to performed to elucidate the mechanisms of how Cyp4a14 is involved in the pathogenesis of cholestatic liver fibrosis.

In conclusion, this study shows a protective effect of Cyp4a14 in cholestatic liver fibrosis through inhibiting HSC activation. The study is the first to elucidate that Cyp4a14 plays a different role in different liver fibrosis model. At present, the reason for the different roles of Cyp4a14 in MCD mice and BDL mice remains obscure. Further study of Cyp4a14 in different fibrosis process might help us better distinguish the different mechanisms behind NASH-related fibrosis and cholestatic-induced liver fibrosis and provide a potential strategy to target and treat clinical liver fibrosis.

## Data Availability Statement

The original contributions presented in the study are included in the article/supplementary material, further inquiries can be directed to the corresponding author.

## Ethics Statement

The animal study was reviewed and approved by the Animal Experimentation Committee of Shenzhen University Health Science Center.

## Author Contributions

SL designed, performed, analyzed, and interpreted the majority of animal experiments and drafted the manuscript. CW and XZ supported the animal experiments. WS designed, planned, and interpreted the study. All authors contributed to the article and approved the submitted version.

## Conflict of Interest

The authors declare that the research was conducted in the absence of any commercial or financial relationships that could be construed as a potential conflict of interest.
